# Bicuspid Valve Sizing for Transcatheter Aortic Valve Implantation: The Missing Link

**DOI:** 10.3389/fcvm.2021.770924

**Published:** 2022-01-27

**Authors:** Giulia Costa, Marco Angelillis, Anna Sonia Petronio

**Affiliations:** Cardiothoracic and Vascular Department, Pisa University Hospital, Pisa, Italy

**Keywords:** aortic stenosis, bicuspid aortic valve, multi-slice computed tomography, sizing, transcatheter aortic valve implantation

## Abstract

Transcatheter aortic valve implantation (TAVI) is a well-recognized and established therapy for severe aortic stenosis, with expanding indications toward younger patients with low surgical risk profile. As bicuspid aortic valve (BAV) affects ~1–2% of the population, it may be speculated that an increasing number of patients with degenerated BAV may eventually need TAVI during the course of the disease. On the other hand, BAV represents a challenge due to its peculiar anatomical features and the lack of consensus on the optimal sizing strategy. The aim of this paper is to review the peculiar aspects of BAV and to discuss and compare the currently available sizing methods. Special attention is given to the role of pre-procedural imaging, mostly with multislice computed tomography, and to the aspects that operators should evaluate in order to ensure an optimal procedural planning and avoid procedural-related complications.

## Introduction

Transcatheter aortic valve implantation (TAVI) has been widely recognized as a safe and effective treatment for aortic stenosis (AS) in patients who cannot undergo surgical aortic valve replacement (SAVR) or are at high or intermediate surgical risk (1–4). Increased operator experience and improved device systems have led to an expanded use of TAVI in lower surgical risk populations (5, 6) and in other pathologies such as bicuspid AS (7, 8). Bicuspid aortic valve (BAV) is the most common congenital cardiac malformation, affecting 1–2% of the population, and is the cause of a significant proportion of aortic valve disease in young adults (9). However, when the progression of the disease is slow, SAVR may be required in older age groups at higher surgical risk due to the age itself and coexistent comorbidities (10, 11). Furthermore, considering the growing expansion of TAVI indications toward younger patients with higher prevalence of bicuspid AS, the clinical outcomes of TAVI in BAV warrant special attention (12). BAV is a challenge for TAVI owing to its complex anatomy with different morphological phenotypes. Peculiar features such as larger dimensions of the aortic valve components, higher calcium burden, presence of a heavily calcified raphe, and associated aortopathy represent some pitfalls when treating BAV patients with TAVI. For these peculiarities and the higher rates of paravalvular leak (PVL), new permanent pacemaker (PPM), need for a second transcatheter heart valve (THV), risk of annulus rupture or aortic dissection, and brain injury (13–15) BAV patients have been initially excluded from the randomized trials. Currently, the use of new-generation devices and the growing attention toward a careful pre-procedural planning have led to an improvement of procedural results, with outcomes nowadays comparable to tricuspid valves (16, 17). However, the unique morphological features of BAV and the lack of consensus on the optimal sizing technique pose a challenge when offering TAVI to such patients.

The aim of this review is to analyze different sizing methods currently used in the real world, taking into account the anatomical features of BAV.

## Anatomical Features

The different morphologies of BAV have been initially classified on the basis of cusps size and number and raphe presence and position. The Sievers and Schmidtke classification (18) divides BAV in three major types: type 0 (no raphe, two leaflets), type 1 (one raphe, fusion of the left coronary cusp with either the right or the non-coronary cusp), and type 2 (two raphes, fusion of the left coronary cusp with both the right and the non-coronary cusp). Whilst this classification was based on the analysis of surgical specimens, in 2014 the BAV Consortium proposed a classification based on transthoracic echocardiography (19). BAVs were classified as type 1 (right-left coronary cusp fusion), type 2 (right-non coronary cusp fusion), and type 3 (left-non coronary cusp fusion). In this classification the raphe can be complete, incomplete or absent. Type 1 BAV without raphe was also indicated as true BAV, corresponding to Sievers' type 0. Finally, Jilaihawi et al. (20) proposed a new classification for BAV based on multi-slice computed tomography (MSCT) imaging, taking in account the increasing role of TAVI in such patients. This new “TAVI-oriented” classification includes three BAV morphologies: tricommissural (the “functional” or “acquired” BAV), bicommissural raphe type, and bicommissural non-raphe type. Leaflet orientation was simplified as coronary cusp fusion or mixed coronary and non-coronary cusp fusion. Interestingly, BAV morphology has been linked to TAVI outcomes, with the presence of a calcified raphe and excessive leaflet calcifications being associated with increased risk of aortic root injury, moderate-to-severe PVL and 30-day mortality (21).

## Bav-Associated Aortopathy

BAV is strongly associated with aortic dilatation and subsequent complications (22), affecting a high percentage of BAV patients and whose pathogenesis is still uncertain. The so-called “BAV-associated aortopathy” has been classified considering the presence or absence of dilatation and the specific location of the aortic disease (23, 24). The different phenotypes have been linked to the presence of either aortic valve stenosis or regurgitation and to the risk of disease progression, with the highest risk related to aortic root dilation (25). Moreover, BAV-associated aortopathy is notoriously associated with an increased risk of aortic dissection compared to the general population, especially with a regurgitant valve (26). The factors that come into play that can affect the development of aortic dilatation in BAV patients are a genetic predisposing milieu (especially mutations involving the TGF-beta signaling pathway) (27) and the chronic hemodynamic overload due to aortic valve disease (28).

## Bav Sizing Techniques

Precise annular sizing based on MSCT imaging is a key step for successful TAVI (29). This is increasingly true in BAV, which displays particular and challenging anatomical characteristics. In BAV, two planes can be identified: the annulus plane and the supra-annular one, where the so-called inter-commissural distance (ICD) is identified. MSCT-based sizing theories take into consideration these two planes, either in a separate or combined fashion. The balloon-sizing method relies on the intra-procedural evaluation of the BAV during predilation. Finally, sizing methods based on the role of the raphe have been proposed.

### “Annular” BAV Sizing

This sizing method is the same used for tricuspid valves, as previously described (30), and identifies the level of the virtual basal ring by connecting the three hinge points at the bottom of the aortic sinuses. The annulus surface area is manually traced, and the geometric mean annulus diameter is then derived. The valve is chosen according the relative sizing chart of each device. The circular shape of the prosthesis is expected to adapt to the aortic annulus, relying on device radial force and on the possibility of raphe fracture. One of the potential limitations of this method is that the unique morphological features of BAV -such as the raphe or the ICD- are not considered. Furthermore, the elliptical shape of the annulus requires some degree of oversizing in order to prevent PVL (31). A possible complication is therefore an excessive oversizing, with increasing risk of annular rupture. This complication is more related to heavily calcified valves or type 1 BAV, where the fibrotic and/or calciphied raphe prevents valve expansion. Yoon et al. (32) in a series of 108 patients treated with balloon-expandable devices reported a rate of 0.9% of annulus rupture and 6.5% of more than moderate PVL. In the experience of Mylotte et al. (33), balloon-expandable valves had a rate of annulus rupture of 0.7%, whereas a more than moderate final PVL was reported in 6% of patients.

### “Supra-Annular” BAV Sizing

To identify the commissures by MSCT, the plane is scrolled in the sagittal view from the annulus to the sinuses, in order to identify the distribution of the leaflets. Then, the position of the commissures is marked and therefore scrolled down to 4 mm above the annulus. The measurement is performed from the middle of one commissure to the middle of the opposite one. The distance of 4 mm has been empirically identified as the reference standard for the measurement of the ICD. The size of the prosthesis is chosen based on the mean perimeter-derived diameter of the annulus and the ICD. The minimal value is used to select the device size, based on the current sizing charts. Currently, this method has been directly investigated only in the BAVARD retrospective registry (34). Briefly, the authors identified three possible aortic configurations: the tubular one, where the mean aortic annulus diameter matches the ICD and can be used for sizing; the flared one, in which the mean aortic annulus diameter is smaller than the ICD; lastly, the tapered one (mean perimeter-derived diameter of the annulus greater than ICD). In this configuration the ICD could be integrated for sizing, as an annulus-based sizing would lead to the selection of a THV too large for the patient. In this registry, annulus-based sizing was applicable to 88% of the patients.

### Annular vs. Supra-Annular Sizing

Randomized comparisons between different sizing methods are currently lacking. In 2019, Kim et al. (35) published a retrospective, single-center analysis of 217 BAV patients treated with TAVI, with annular sizing being the default method for all patients. Overall, no significant differences where found between ICD and annulus measurements, despite some intra-individual differences. Supra-annular sizing would have resulted in a divergent size selection in more than one-third of patients. On the basis of these results, the authors concluded that supra-annular sizing might have a role in few cases with annular sizing errors, but might also lead to improper THV selection in a considerable percentage of patients. Accordingly, Weir-McCall et al. (36) analyzed a series of 44 patients treated with balloon-expandable THV. Annulus-based device sizing displayed substantial agreement with the chosen THV, whereas a much weaker reproducibility was obtained by supra-annular sizing (performed by generating a circle defined by the ICD).

Overall, supra-annular sizing methods are widely varied, with no consistent recommendation on which height(s) to measure and no consistent tools or techniques on how to measure. Moreover, there is a lack of prospective evidence comparing clinical results to basal plane annulus measurement results.

### “Balloon-Technique” BAV Sizing

When the virtual ring measurements falls into a borderline range, the correct prosthesis sizing might be assessed referring to the relationship between the inflated balloon during balloon pre-dilatation and the Valsalva sinuses. A pigtail catheter is placed at the bottom of the right coronary cusp and the C-arm is moved until the coaxial implantation view is obtained using the described “right cusp rule” (37). A contrast injection is performed to achieve optimal visualization of the three cusps, assuming that the distance between the non-coronary and left cusp hinge points correlates to the annulus diameter. A balloon is placed across the virtual aortic annulus and fully expanded under rapid pacing. If the balloon reaches the hinge points, the size of balloon corresponds to the valve size. Otherwise, a larger THV can be chosen. Moreover, the presence of contrast backflow into the left ventricle during valvuloplasty or excessive movement of the inflated balloon suggests that there is insufficient coverage of the annulus, and therefore that a larger THV is recommended. While this method is easy to apply in tricuspid valves, it can be more challenging in BAV because of the asymmetrical distribution of the cusps.

In 2018, Liu et al. (38) described a sizing method for BAV using balloon pre-dilatation based on the so-called “waist sign.” Sequential balloon aortic pre-dilatations beginning with the smaller size of 18 mm were performed, with 2 mm increments until the “waist sign” occurred with less than mild regurgitation during contrast injection. This method has been used in a case series of 12 patients obtaining a downsizing of the THV compared to the MSCT in 91.7% of patients. No aortic ruptures and no residual moderate or severe PVL were reported.

### “Raphe-Based” Sizing

**Figure 1:**
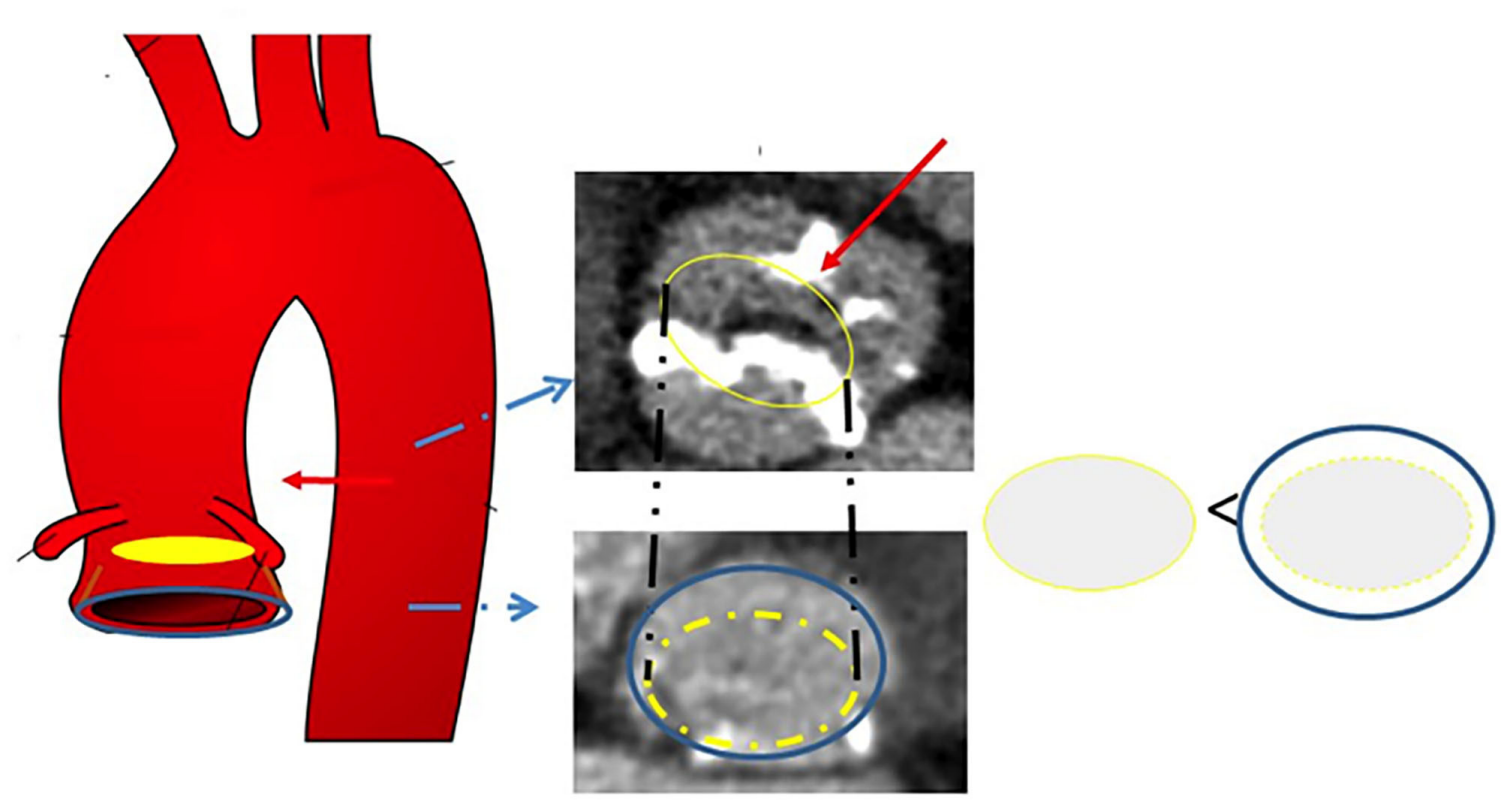
Schematic representation of the effects of the raphe on THV accommodation. The blue circle indicates the annulus, whereas the yellow circle represents the ideal space at the level of the raphe able to accommodate the prosthesis. THV, transcatheter heart valve.

**Figure 2:**
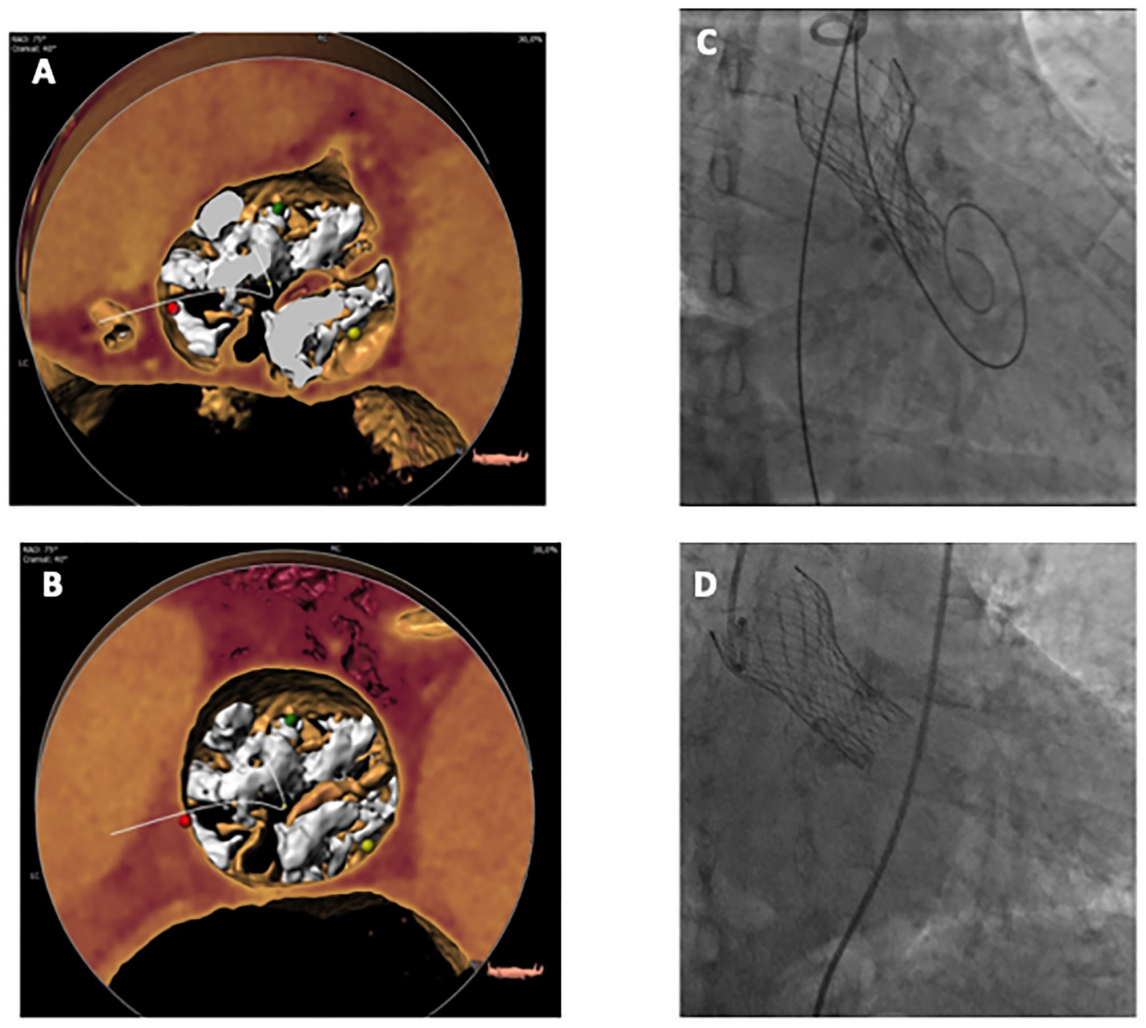
3D Volume Rendering MSCT view of a BAV at annulus level **(A)** and raphe level **(B)**, in this case at 10.4 mm from annulus plane. Constrained THV right after release **(C)** and final result after post-dilatation **(D)**. BAV, bicuspid aortic valve; MSCT, multislice computed tomography; THV, transcatheter heart valve.

These methods are based on the assumption that the raphe plays a pivotal role in the accommodation of the THV in BAV, as this structure is often stiff and heavily calcified, impairing proper valve expansion. Figures 1, 2 display the impact of the raphe on the actual space able to accommodate the THV.

### Casper Algorithm

Recently, we proposed a new algorithm for BAV sizing (39). This algorithm takes into account both the annulus plane and the supra-annular one. It has been developed from MSCT measurements before and after TAVI in a series of patients with type 1 BAV. This algorithm is based on three main factors: (1) Raphe length is related to incomplete valve expansion; (2) Calcium burden and distribution are associated with lower valve expansion; (3) Raphe length is the most reliable measure with high inter-observer reproducibility. Starting from the annulus-derived diameter, several millimeters (up to 2.5) are subtracted taking into account the presence of heavy calcium burden, raphe length and calcium distribution. This method has been applied to 21 patients, obtaining 100% procedural success and excellent THV performance.

### Lira Method

The method described by Iannopollo et al. (40) aims to recognize the plane where valve anchoring is assumed. For BAV type 1, the prosthesis should anchor at the level of the raphe. Therefore, the LIRA plane is identified as the plane that encounters the raphe at its maximum protrusion. In type 2 BAV, the prosthesis should anchor at the level of the major raphe -defined as the larger one, with the greater amount of calcium. The LIRA plane represents a “neo-virtual basal ring” where the perimeter traces the internal border of the leaflets, excluding all the structures encountered at this level (fused commissures, heavy calcification, etc.). This sizing method has been evaluated in a cohort of 20 patients, with excellent THV performance.

Table 1 resumes the principal characteristics of the currently available sizing methods.

**Table 1:** Principal characteristics of the currently available sizing methods for BAV.

	**Annular sizing**	**Supra-annular sizing**	**Balloon sizing**	**Raphe-based sizing**
Undersizing	No	Yes/no	Yes/no	Yes/no
Raphe evaluation	No	No	Yes/no	Yes
Applicability to all THV	Yes	Yes	Yes	Yes
Reproducibility	Yes	Yes/no	No	Yes
Calcium evaluation	No	No	No	Yes

## Discussion and Conclusions

BAV is improperly believed to be a relatively rare aortic valve abnormality, with BAV type I as the most frequent subtype. Nowadays, a higher number of AS patients with BAV are reported. This increasing trend can be also related to the extensive use of MSCT in pre-procedural planning. Recent studies and registries described encouraging results in this subset of patients, demonstrating that newer generation THV can offer better results than the first generation ones (8, 12, 14, 16, 17, 41). As a consequence, the balloon-expandable (Sapien 3™, Edwards Lifesciences, CA, USA) and self-expandable (Evolut R/PRO™, Medtronic, MN, USA) THV have received US Food and Drug Administration and European Conformity approval for all categories of surgical risk regardless of anatomy.

Nevertheless, this abnormal morphology in aortic stenosis entails possible complications such as higher rate of new PPM, moderate to severe PVL, prosthesis embolization and annulus rupture. As BAV is more frequent in younger patients, the foreseen utilization of TAVI in these patients necessitates a need for consensus on procedural and device planning. The strong association between BAV and aortopathy may pose a challenge when addressing such patients to TAVI, considering the risk of progression of the aortic disease. Even if robust data on the evolution of aortic disease in BAV patients treated with TAVI are currently lacking, data from surgical series show that the correction of hemodynamic overload may slow the progression of the disease (28); this might be extrapolated and applied also to TAVI patients. Moreover, a small study on 67 BAV patients treated with TAVI and with aortic diameter <50 mm showed no significant progression of the aortic disease at a median follow-up of 398 days (42). It is as well uncertain if BAV-associated aortopathy may lead to an increased risk of aortic injury during TAVI; with this in mind, the use of flexible devices and avoidance of prosthesis oversizing and aggressive dilation may be advocated.

The different features in BAV compared to tricuspid aortic valves and the variety of subtypes rise the question if BAV have to be measured like tricuspid valves or if a new method of measurement is warranted. As the choice of the THV size is modulated by the presence or absence of some features, instead of referring to sizing techniques such as oversizing or undersizing operators should aim to a *tailored* sizing. The different techniques that have emerged have not been yet tested in large series of patients, and therefore it appears mandatory to better understand the correct method for BAV sizing. It should be underlined that different devices might need different sizing techniques. Currently, data on THV durability in the setting of BAV are lacking, despite encouraging results from early and mid-term outcomes (16, 17). In BAV, some degree of asymmetry is expected due to the presence of raphe and heavy calcifications. As eccentricity and non-circularity have been related to unfavorable valve hemodynamics and a theoretic impact on THVs durability (43), correct sizing is of paramount importance in this setting, especially in younger populations.

Several registries are currently investigating the results of different sizing methods. The BIVOLUT X registry (Bicuspid aortic stenosis with Evolut platform international experience, NCT03495050) is the first international registry of BAV with the attempt to evaluate different sizing methods by means of an imaging-based approach using a pre and a post-MSCT analysis. Moreover, the CASPER registry (NCT04817735) is currently investigating the safety and efficacy of BAV sizing based on calcium burden and raphe length (the CASPER algorithm). Larger, prospective, and randomized trials are expected in order to evaluate mid and long-term follow up of these patients, with possible comparison between SAVR and TAVI results in this setting.

BAV represents a challenge for TAVI operators, both for its peculiar anatomic features and because the progressive shift toward younger patients with low surgical risk and high life expectancy, where optimal procedural results are expected. Careful pre-procedural planning and standardized sizing methods are warranted in order to guarantee a tailored approach and the best possible outcomes.

## Author Contributions

GC, MA, and AP equally contributed in conception and design of the manuscript, drafting of the manuscript or revising it critically for important intellectual content, and final approval of the manuscript submitted. All authors contributed to the article and approved the submitted version.

## Conflict of Interest

AP is a consultant and received fund from Medtronic, Boston and ABBOTT. The remaining authors declare that the research was conducted in the absence of any commercial or financial relationships that could be construed as a potential conflict of interest.

## Publisher's Note

All claims expressed in this article are solely those of the authors and do not necessarily represent those of their affiliated organizations, or those of the publisher, the editors and the reviewers. Any product that may be evaluated in this article, or claim that may be made by its manufacturer, is not guaranteed or endorsed by the publisher.

## Abbreviations

AS: aortic stenosis
BAV: bicuspid aortic valve
MSCT: multi-slice computed tomography
PVL: paravalvular leak
TAVI: transcatheter aortic valve implantation
THV: transcatheter heart valve.
